# Kidney Injury Following Ibuprofen and Acetaminophen: A Real-World Analysis of Post-Marketing Surveillance Data

**DOI:** 10.3389/fphar.2021.750108

**Published:** 2021-12-22

**Authors:** Qi-hui Shao, Xue-dong Yin, Hong-xia Liu, Bin Zhao, Jian-quan Huang, Zhi-ling Li

**Affiliations:** ^1^ Department of Pharmacy, Shanghai Children’s Hospital, Shanghai Jiao Tong University, Shanghai, China; ^2^ Shanghai Jiao Tong University School of Medicine, Shanghai, China; ^3^ Pharmacy Department, Peking Union Medical College Hospital, Peking Union Medical College, Chinese Academy of Medical Sciences, Beijing, China

**Keywords:** ibuprofen, acetaminophen, adverse event reporting system, epidemiology, kidney injury

## Abstract

**Background:** Although kidney injury has been reported as a serious adverse effect in patients treated with ibuprofen or acetaminophen (APAP), there are still few real-world studies to compare the specific differences in the adverse effects of nephrotoxicity.

**Methods:** Disproportionality analysis and Bayesian analysis were devoted to data-mining of the suspected kidney injury after using ibuprofen and APAP based on the FDA’s Adverse Event Reporting System (FAERS) from January 2004 to March 2021. The times to onset, fatality, and hospitalization rates of ibuprofen-associated kidney injury and APAP-associated kidney injury were also investigated.

**Results:** 2,453 reports of ibuprofen-associated kidney injury and 1,288 reports of APAP-associated kidney injury were identified. Ibuprofen appeared to affected more middle-aged patients than elderly ones (27.76 vs 16.53%) while APAP appeared to affected more young patients than middle-aged patients (45.24 vs 29.10%) and elderly patients were fewer (13.99%). Compared to ibuprofen, APAP had the higher association with renal injury based on the higher reporting odds ratio (ROR = 2.45, 95% two-sided CI = 2.36–2.56), proportional reporting ratio (PRR = 2.39, *χ* ^2^ = 2002.94) and empirical Bayes geometric mean (EBGM = 2.38, 95% one-sided CI = 2.3). In addition, APAP-associated kidney injury had earlier onset (32.74 vs 115.82 days, *p* < 0.0001) and a higher fatality rate (44.43 vs 7.36%, *p* < 0.001) than those of ibuprofen-associated kidney injury.

**Conclusion:** The analysis of FAERS data provides a more accurate profile on the incidence and prognosis of kidney injury after ibuprofen and acetaminophen treatment, enabling continued surveillance and timely intervention in patients at risk of kidney injury using these drugs.

## Introduction

Ibuprofen, a propionic acid derivative, and acetaminophen, an aniline derivative, are nonsteroidal anti-inflammatory drugs (NSAIDs). NSAIDs inhibit cyclooxygenase (COX) to reduce prostaglandin (PG) production, so that the PG-mediated inflammatory response can be weakened (Giménez-Bastida et al., 2019). As first-line antipyretic and analgesic drugs, they have been widely used for a long time, not only as prescription drugs but also as over-the-counter (OTC).

In the past, misconceptions about their safety led to the randomness of drug use. Long-term drug use or overdose has produced a series of adverse reactions, severe cases can be life-threatening. Analysis showed that 56,000 emergency department visits, 26,000 hospitalizations and 458 deaths were attributed to acetaminophen-associated overdoses in the United States from 1991 to 1998 (Nourjah et al., 2006). As people’s understanding of drugs has improved in the last decades, the number of adverse outcomes caused by ibuprofen and acetaminophen has decreased significantly (Tan et al., 2020). Among these serious adverse reactions, renal function damage cannot be ignored.

Since 1946, there have been studies of analgesic nephropathy (KOLFF, 1946), which refers to chronic renal tubulointerstitial nephropathy and renal papillary necrosis caused by the long-term and large amount of NASIDs and their compound preparations (Liu and Shen, 2009). However, most of the evidence for acetaminophen- or ibuprofen-associated kidney injury came from case reports and clinical trials, so it is significant to update our understanding and outline the risks and characteristics of adverse events after ibuprofen and acetaminophen treatment for further prevention and management. Therefore, we attempted to evaluate and compare the association between ibuprofen, acetaminophen, and kidney injury in a large population by investigating the FDA’s Adverse Event Reporting System (FAERS). Meanwhile, the differences in onset time and mortality between ibuprofen-associated kidney injury and APAP-associated kidney injury were further investigated.

## Materials and Methods

### Data Source

A retrospective pharmacovigilance study was conducted using data retrieved from the FAERS database from January 2004 and March 2021. 3,673 reports of ibuprofen-associated kidney injury and 2,296 reports of APAP-associated kidney injury were retrieved from the FAERS database in total and deduplicated records were removed according to the FDA’s recommendations. Finally, 2,453 reports of ibuprofen-associated kidney injury and 1,288 reports of APAP-associated kidney injury were identified.

### Adverse Event and Drug Identification

We investigated adverse events by using the MedDRA (Version 24.0) Preferred Terms as follows: acute kidney injury [10069339], subacute kidney injury [10081980], acute prerenal failure [10001017], renal failure acute ischemic [10038439], blood creatinine increased [10005483], blood urea abnormal [10005846], glomerular filtration rate decreased [10018358], renal impairment [10062237], oliguria [10030302], anuria [10002847], dialysis [10061105], proteinuria [10037032], nephrotic osmotic [10029163], renal tubular injury [10078933], nephropathy toxic [10029155], nephritis allergic [10029120], tubulointerstitial nephritis [10048302]. Thus, the MICROMEDEX^®^ (Index Nominum) was used like a dictionary. Ibuprofen and acetaminophen were defined as both brand and generic names in the DRUG file, and the role of the drug was identified as primary suspected.

### Data Mining

Based on the basic principles of Bayesian analysis and non-proportional analysis, we applied the reporting odds ratio (ROR), the proportional reporting ratio (PRR), the Bayesian confidence propagation neural network (BCPNN) and the multi-item gamma Poisson shrinker (MGPS) algorithms to investigate the association between ibuprofen or APAP and the adverse reactions. The equations and criteria for the four algorithms (DuMouchel, 1999; Evans et al., 2001; Szarfman et al., 2002; van Puijenbroek et al., 2002; Hauben, 2003; Hauben et al., 2005; Norén et al., 2006; Ooba and Kubota, 2010; Szumilas, 2010) are shown in Table 1. These algorithms were extracted to measure the strength of the association between drugs and adverse events, and if one of the four algorithms met the criteria, it should be considered a positive signal for kidney injury.

**TABLE 1:** Summary of major algorithms used for signal detection.

Algorithms	Equation*	Criteria
ROR	ROR = (a/b)/(c/d)	95% CI > 1, *N* ≥ 2
	95%CI = e^ln(ROR) ± 1.96(1/a+1/b+1/c+1/d)^0.5^	
PRR	PRR = [a/(a + c)]/[b/(b + d)]	PRR ≥ 2, *χ* ^2^ ≥ 4, *N* ≥ 3
	*χ* ^2^ = Σ[(O − E)^2^/E]; [O = a, E = (a + b)(a + c)/(a + b + c + d)]	
BCPNN	IC = log_2_a (a + b + c + d)/[(a + c)(a + b)]	IC025 > 0
	IC025 = e^ln(IC)−1.96(1/a+1/b+1/c+1/d)^0.5^	
MGPS	EBGM = a (a+b + c + d)/[(a + c)(a + b)]	EB05 ≥ 2, *N* > 0
	EB05 = e^ln(EBGM)−1.64(1/a+1/b+1/c+1/d)^0.5^	

*a: number of reports containing both the suspect drug and the suspect adverse drug reaction. b: number of reports containing the suspect adverse drug reaction with other medications (except the drug of interest). c: number of reports containing the suspect drug with other adverse drug reactions (except the event of interest). d: number of reports containing other medications and other adverse drug reactions. Abbreviations: ROR, reporting odds ratio; CI, confidence interval; N, the number of co-occurrences; PRR, proportional reporting ratio; χ^2^.

Chi-squared; BCPNN, Bayesian confidence propagation neural network; IC, information component; IC025, the lower limit of the 95% two-sided CI, of the IC; MGPS, multi-item gamma Poisson shrinker; EBGM, empirical Bayesian geometric mean; EB05, the lower 90% one-sided CI, of EBGM.

We calculated the onset time of kidney injury following ibuprofen and acetaminophen respectively, which was defined as the interval between EVENT_DT (adverse event onset date) and START_DT (start date of ibuprofen or APAP administration). Reports with incorrect input (EVENT_DT before START_DT) or incorrect data input were also excluded. In addition, mortality would be defined as the number of fatal events divided by the total number of ibuprofen- or acetaminophen-related kidney injuries.

### Statistical Analysis

Descriptive analysis was applied to summarize the clinical characteristics of kidney injury patients resulted in ibuprofen and APAP from the FAERS database. The Mann-Whitney test was used to compare the time to onset of ibuprofen-associated kidney injury and APAP-associated kidney injury. Pearson’s chi-square test or Fisher’s exact test was utilized to compare the mortality and hospitalization rates between ibuprofen and APAP. The statistical significance was set at *p* < 0.001 with 95% confidence intervals. All statistical analyses were performed using GraphPad Prism 8 (GraphPad Software, CA, United States).

## Results

### Disproportionality Analysis and Bayesian Analysis

From January 2004 to March 2021, a total of 7,411 cases of kidney injury-related reports were recorded in the FAERS database. A total of 2,453 cases of kidney injury induced by ibuprofen as a suspicious drug and 1,288 cases of kidney injury induced by APAP as a suspicious drug were identified. According to the standards of the four algorithms, the renal injury signals were detected for ibuprofen and acetaminophen. As shown in Table 2, both ibuprofen and acetaminophen have statistically significant ROR, PRR and information component (IC), while only acetaminophen has statistically significant empirical Bayesian geometric mean (EBGM).

**TABLE 2:** Signal detection for ibuprofen-associated kidney injury and APAP-associated kidney injury.

Drugs	*N*	ROR	PRR	IC	EBGM
		(95% two-sided CI)	(*χ* ^2^)	(IC025)	(EBGM05)
Ibuprofen	1,287	1.34 (1.27,1.41)*	1.33 (106.38)*	0.41 (0.39)*	1.33 (1.27)
APAP	2,451	2.45 (2.36,2.56)*	2.39 (2002.94)*	1.25 (1.20)*	2.38 (2.30)*

APAP, acetaminophen; ROR, reporting odds ratio; CI, confidence interval; PRR, proportional reporting ratio; *χ* ^2^, chi-squared; IC, information component; EBGM, empirical Bayesian geometric mean; *statistically significant.

### Descriptive Analysis

The clinical features were summarized in Table 3. Except for the unspecified age, the renal injury was more likely to occur in middle-aged patients treated with ibuprofen than the elderly patients (27.76 vs 16.53%), and young patients (18–44 years old) accounted for 22.31% reported cases. However, young patients treated with APAP were more affected than middle-aged patients (45.24 vs 29.10%), and patients elder than 65-year-old only accounted for 13.99% reported cases. Except for the unspecified data, in the case of APAP, females made up more reports than males (55.90 vs 44.10%) while in the case of ibuprofen, the proportion of females and males were almost equal (50.02 vs 49.98%). In terms of ibuprofen, about two-thirds of the reports were from Europe (61.15%), and about one-third of the reports were from North America (32.37%). In terms of APAP, nearly half of the reports were from North America (47.44%), followed by 36.80% of reports from Europe. For both ibuprofen and acetaminophen, health-professional submitted most of the reported cases (ibuprofen: 75.54%, APAP: 75.47%), and among them, other health-professional who were not the pharmacist or the physician accounted for the majority (ibuprofen: 32.57%, APAP: 39.52%).

**TABLE 3:** Clinical characteristics of patients with ibuprofen-related kidney injury and APAP-related kidney injury collected from the FAERS database (December 2003 to March 2021).

Characteristics	Reports (*N*, %)	
	Ibuprofen	Acetaminophen
Patient age (year)		
<18	431 (17.57)	125 (9.70)
18–44	459 (18.71)	485 (37.66)
45–64	571 (23.28)	312 (24.22)
65–74	340 (13.86)	68 (5.28)
>74	256 (10.44)	82 (6.37)
Unknown	396 (16.14)	216 (16.77)
Patient gender		
Female	1,073 (43.74)	635 (49.30)
Male	1,072 (43.70)	501 (38.90)
Unknown	308 (12.56)	152 (11.80)
Area		
Africa	2 (0.08)	1 (0.08)
Asian	60 (2.45)	93 (7.22)
Europe	1,500 (61.15)	474 (36.80)
Oceania	36 (1.47)	17 (1.32)
North America	794 (32.37)	611 (47.44)
South America	10 (0.41)	1 (0.08)
Unknown	51 (2.08)	91 (7.07)
Reporters		
Consumer	152 (6.20)	55 (4.27)
Lawyer	4 (0.16)	2 (0.16)
Pharmacist	364 (14.84)	91 (7.07)
Physician	690 (28.13)	372 (28.88)
Other health-professional	799 (32.57)	509 (39.52)
Unknown	444 (18.10)	259 (20.11)
Outcome event		
Death	178 (7.36)	570 (44.43)
Hospitalization-Initial or Prolonged	1,778 (73.50)	838 (65.32)
Disability	37 (1.53)	13 (1.01)
Life-Threatening	323 (13.35)	205 (15.98)
Other Serious (Important Medical Event)	1,327 (54.86)	652 (50.82)
Required Intervention to Prevent Permanent Impairment/Damage	28 (1.16)	17 (1.33)
Congenital Anomaly	2 (0.08)	1 (0.08)

FAERS, FDA adverse event reporting system; APAP, acetaminophen.

**FIGURE 1:**
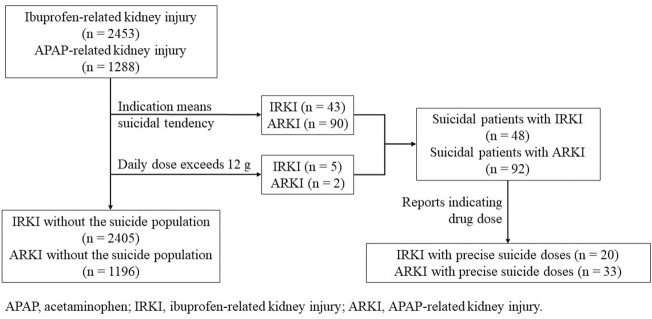
Process of the selection of different groups of ibuprofen- and acetaminophen-related kidney injury.

Meanwhile, considering that the suicide rate of OTC analgesics increased significantly by 33.5% from 2000 to 2018 (Hopkins et al., 2020), we eliminated all reports that used suicidal attempt as the purpose of medication (Figure 1). We believed it was the suicidal population that the indication of the report meant that people who were suicidal or whose daily dose exceeded 12 g (Tallarida, 1982; Pourarian et al., 2015; Fox et al., 2012). The results before and after the suicide data were removed did not change. In addition, we further screened out reports that indicated the dosage of the drug. A total of 20 ibuprofen-related suicide reports (Table 4) and 33 acetaminophen-related suicide reports were obtained (Table 5). For ibuprofen, all reported doses were no less than 1.2 g, among which 11 cases were no less than 12 g, and even three cases used a dose of about 100 g. The median dose used was 12 g, which was 10 times the upper limit of normal dose (1.2 g). Among the 20 cases reported, there were 13 females, far more than males. People under the age of 18 were most susceptible (eight cases). A total of 13 patients had an adverse outcome (hospitalization or death), of which seven patients received doses exceeding 12 g. For acetaminophen, all reported doses were no less than 2 g, among which 23 cases were no less than 16 g. The median dose used was 28 g, which was about seven times the upper limit of normal dose (3.9 g). Among the 33 cases reported, there were 21 females. Middle-aged people (45–64 years old) were most likely to be suicidal using acetaminophen (16 cases). Life-threatening was the most common outcome (11 cases). A total of 12 patients had adverse outcomes, of which eight cases received a dose of 16 g or more.

**TABLE 4:** The relationship between suicide with ibuprofen and dose (December 2003 to March 2021).

Indication	Dose (g)	Outcome
Intentional overdose	2.4	Life-Threatening
Poisoning deliberate	9.6	Hospitalization—Initial or Prolonged
Poisoning deliberate	9.6	Hospitalization—Initial or Prolonged
Poisoning deliberate	9.6	Hospitalization—Initial or Prolonged
Poisoning deliberate	9.6	Hospitalization—Initial or Prolonged
Suicidal ideation	6.4	Hospitalization—Initial or Prolonged
Suicidal ideation	6.4	Hospitalization—Initial or Prolonged
Suicide attempt	12	Other Serious (Important Medical Event)
Suicide attempt	12	Hospitalization—Initial or Prolonged
Suicide attempt	23	Hospitalization—Initial or Prolonged
Suicide attempt	23	Life-Threatening
Suicide attempt	8	Life-Threatening
Suicide attempt	8	Life-Threatening
Suicide attempt	12.4	Hospitalization—Initial or Prolonged
Overdose	28.8	Hospitalization—Initial or Prolonged
Unknown	95.4	Hospitalization—Initial or Prolonged
Unknown	12	Other Serious (Important Medical Event)
Unknown	105	Death
Unknown	24	Other Serious (Important Medical Event)
Unknown	100	Hospitalization—Initial or Prolonged

**TABLE 5:** The relationship between suicide with acetaminophen and dose (December 2003 to March 2021).

Indication	Dose (g)	Outcome
Intentional overdose	50	Hospitalization—Initial or Prolonged
Intentional overdose	40	Hospitalization—Initial or Prolonged
Intentional overdose	30	Hospitalization—Initial or Prolonged
Intentional overdose	15	Other Serious (Important Medical Event)
Intentional product misuse	40	Other Serious (Important Medical Event)
Poisoning deliberate	40	Life-Threatening
Poisoning deliberate	40	Life-Threatening
Poisoning deliberate	16	Life-Threatening
Poisoning deliberate	16	Life-Threatening
Poisoning deliberate	16	Life-Threatening
Poisoning deliberate	16	Life-Threatening
Poisoning deliberate	8	Hospitalization—Initial or Prolonged
Poisoning deliberate	8	Hospitalization—Initial or Prolonged
Poisoning	28	Other Serious (Important Medical Event)
Poisoning	28	Life-Threatening
Suicide attempt	40	Other Serious (Important Medical Event)
Suicide attempt	40	Other Serious (Important Medical Event)
Suicide attempt	90	Hospitalization—Initial or Prolonged
Suicide attempt	3	Death
Suicide attempt	3	Death
Suicide attempt	16	Hospitalization—Initial or Prolonged
Suicide attempt	30	Other Serious (Important Medical Event)
Suicide attempt	30	Other Serious (Important Medical Event)
Suicide attempt	3	Other Serious (Important Medical Event)
Suicide attempt	2	Other Serious (Important Medical Event)
Suicide attempt	8	Life-Threatening
Suicide attempt	8	Other Serious (Important Medical Event)
Suicide attempt	20	Life-Threatening
Suicide attempt	7	Life-Threatening
Overdose	50	Death
Overdose	27	Hospitalization—Initial or Prolonged
Unknown	50	Hospitalization—Initial or Prolonged
Unknown	28	Life-Threatening

### Time to Onset of Ibuprofen- and APAP-Associated Renal Injury

**FIGURE 2:**
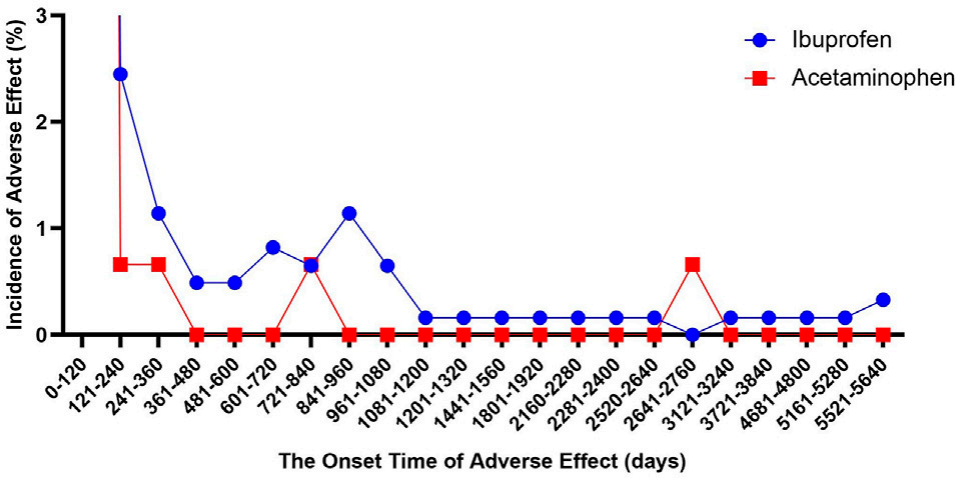
The onset time of ibuprofen- and acetaminophen-associated renal injury (December 2003 to March 2021).

We described the time to onsets of renal events for ibuprofen and APAP in Figure 2. According to the data, the median onset time of acetaminophen-related kidney injury was 2 days [interquartile range (IQR) 0–7], and the median onset time of ibuprofen-related kidney injury was 5 days (IQR 2–20). Besides, there was a significant difference in average time to onset of renal events among ibuprofen and APAP (Mann-Whitney test, *p* < 0.0001). The average onset time of APAP-related kidney injury was 32.74 days, which was about a quarter of that of ibuprofen-related kidney injury (115.82 days).

Similarly, we studied the onset time of kidney injury in all patients who used these two drugs to commit suicide, and found that they were all concentrated within 120 days. The median time to onset of ibuprofen-related kidney injury did not change, and the median time to onset of acetaminophen-related kidney injury was extended back by 1 day (3 days, IQR 0–8). There was also a significant difference in average time to onset of renal events among ibuprofen (118.71 days) and APAP (38.86 days) (Mann-Whitney test, *p* < 0.0001).

### Fatality and Hospitalization due to Ibuprofen-and APAP-Associated Kidney Impairment

The rate of fatality and hospitalization due to renal injury following ibuprofen and acetaminophen were assessed to analyze the prognosis of ibuprofen- and APAP-associated kidney injury. The hospitalization rate of ibuprofen-associated renal injury was 73.50%, and that of acetaminophen-associated renal injury was 65.32%, yet the mortality rate of kidney injury caused by ibuprofen is much lower than that caused by acetaminophen (7.36 vs 44.43%) and significant differences in both hospitalization rate and mortality rate were found between ibuprofen and acetaminophen (Fisher’s exact test, *p* < 0.0001). After removing patients with suicidal tendencies, the data only slightly changed (Hospitalization: 73.98 vs 68.18%; Fatality: 7.09 vs 44.67%), and the results were not inconsistent (Fisher’s exact test, *p* < 0.0001).

### Complications of Ibuprofen- and Acetaminophen-Related Renal Injury

**FIGURE 3:**
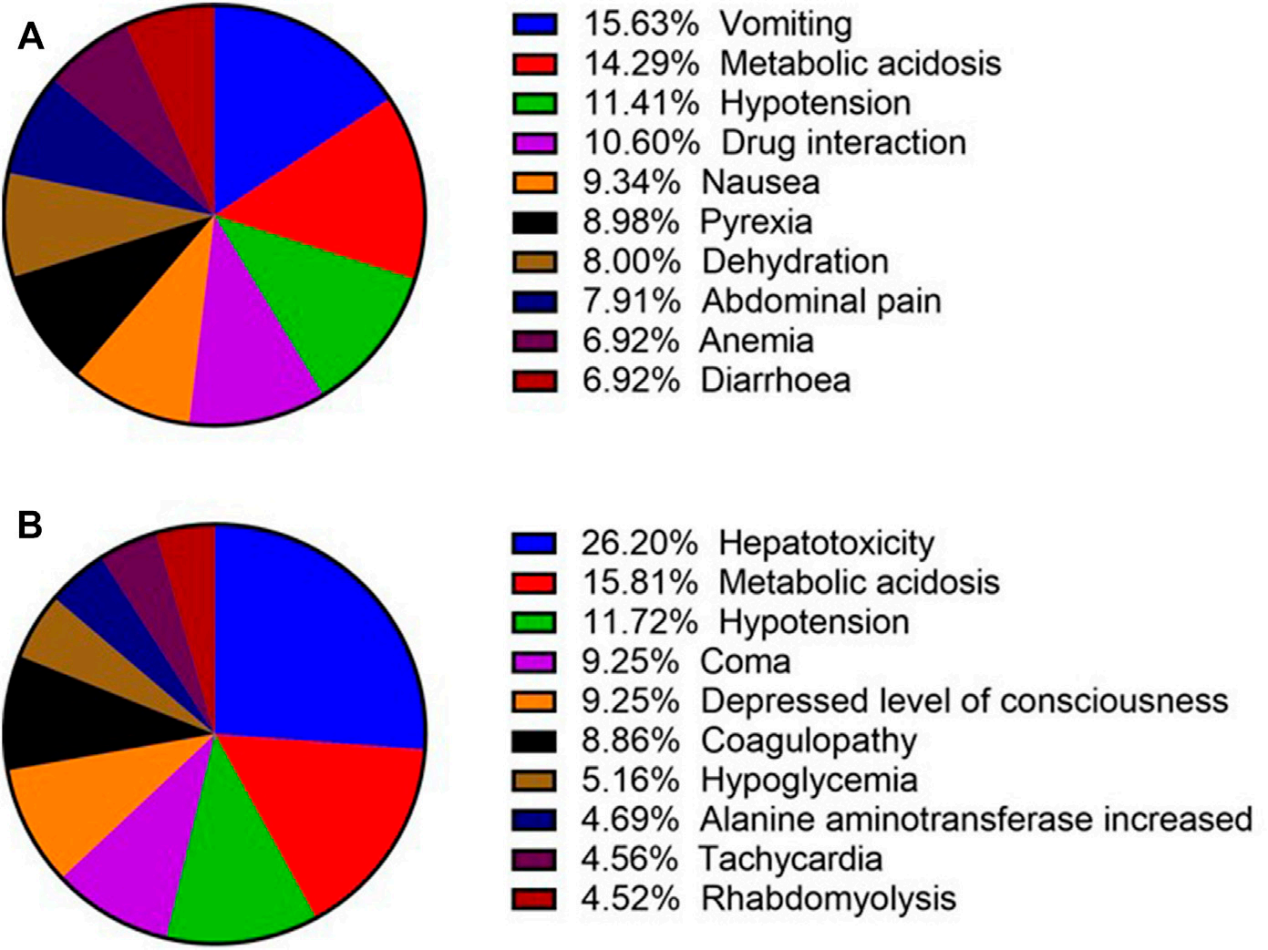
Top 10 complications of ibuprofen- and acetaminophen-related renal injury (December 2003 to March 2021). **(A)** Top 10 complications of ibuprofen-related renal injury; **(B)** Top 10 complications of acetaminophen-related renal injury.

We searched for the complications of ibuprofen- and acetaminophen-related kidney injury, and selected the top ten complications (Figure 3). In terms of ibuprofen-related kidney injury, vomiting ranked first with the number of 174, which exceeded metabolic acidosis 15 cases. Hypotension ranked third with 127 reports. In terms of acetaminophen-related kidney injury, the number of hepatotoxicity cases is about 1.7 times the number of cases of metabolic acidosis, ranking first. Hypotension also ranks third.

## Discussion

To the best of our knowledge, this study is the first and largest collection to describe differences in the vulnerable population, onset time and adverse outcomes of kidney injury following ibuprofen and acetaminophen in real-world practice based on the FAERS pharmacovigilance database from January 2004 to March 2021. All previous related studies have a small sample size (<16 cases) (Dixit et al., 2010; Waring et al., 2010), or only focus on groups with a certain disease background or a certain surgical operation (Kandler et al., 2014; Van Driest et al., 2018). Thus, they did not discuss the characteristics of kidney injury induced by the two drugs from a more comprehensive perspective, nor have they compared the kidney injury induced by the two drugs, especially the onset time.

Multiple studies have demonstrated the relationship between acute kidney injury (AKI) and ibuprofen monotherapies and combination therapies in patients with different geographical, age, and underlying conditions (Balestracci et al., 2015; Salerno et al., 2021; Su et al., 2021). Meanwhile, acetaminophen overuse is associated with AKI in approximately 12% of patients (Akakpo et al., 2020a), and a study found that ibuprofen was more likely than acetaminophen to increase the risk of AKI when used for preoperative PDA closure (Balachander et al., 2020). It is worth noting that the current definition of AKI is mainly based on the risk, injury, failure, loss, end-stage kidney disease (RIFLE) criteria, the acute kidney injury network (AKIN) criteria and the kidney disease improving global outcome (KDIGO) criteria (Koza, 2016). Although the criteria selected in the various studies mentioned above are different, these criteria all have similar ability to predict in-hospital mortality (Er et al., 2020). Therefore, this may have a slight impact on the data on the incidence of ibuprofen- and acetaminophen-associated AKI.

Although the association between ibuprofen or acetaminophen and AKI has been reported in many literatures, little has been reported in chronic kidney disease. Real world analyses of post-market surveillance are even less. Based on the network of the Berlin Case-Control Surveillance Study, 143 patients with drug-induced kidney injury from April 2010 to December 2011 were included and it was concluded that NSAIDs seemed to exhibit nephrotoxicity even when baseline renal function of patients was normal (Douros et al., 2018). In the case of acetaminophen, the relationship between long-term analgesic nephropathy and APAP remains unexplored (McCrae et al., 2018).

### Mechanisms of Ibuprofen- and APAP-Related Kidney Injury

The mechanisms by which ibuprofen and acetaminophen cause kidney injury are different. Ibuprofen produces nephrotoxicity in three ways. One is the direct toxicity to the kidney. The second is secondary renal damage caused by the deposition of antigen-antibody complexes (Ag-Ab complexes) formed by ibuprofen on the glomeruli. The third is determined by its pharmacological effects. Ibuprofen inhibits COX enzyme to reduce PG production so that the renal tubules contract, resulting in a decrease in renal blood flow (RBF), a decrease in glomerular filtration rate (GFR), and finally renal tubular toxicity (Li, 2001). The mechanism of acetaminophen-associated kidney injury is still controversial. Possible reasons include the local production of N-acetyl-p-benzoquinonimine (NAPQI) or other toxic metabolites such as p-aminophenol by CYP or COX enzymes (Carpenter and Mudge, 1981; Bessems and Vermeulen, 2001). Studies have found that the conjugate of glutathione or cysteine and NAPQI can act as a γ-glutamyl receptor substrate and specifically depletes glutathione in the kidney, so that more free NAPQI binds to cellular proteins, and further aggravates nephrotoxicity (Stern et al., 2005a; Stern et al., 2005b). However, the consumption of glutathione is not the only cause of nephrotoxicity (Eguia and Materson, 1997). In addition, cell apoptosis involved in APAP-related nephrotoxicity is mainly regulated by the endoplasmic reticulum (ER) pathway (Lorz et al., 2004).

### Descriptive Analysis

According to epidemiological results, 56.74% of ibuprofen-related kidney injury was detected in middle-aged and elderly people, and 74.34% of APAP-related kidney injury was detected in middle-aged and young people. This may be related to the different mechanisms of nephrotoxicity between the two. The renal parenchyma of middle-aged and elderly people may have atrophied to a certain extent, and then the RBF and GFR have decreased (Lucas et al., 2019), coupled with the deposition of the Ag-Ab complexes of ibuprofen, may lead to further deterioration of renal function, and ultimately lead to kidney injury. The age bias of APAP has also been found in other studies (Chen et al., 2015), which attributed the difference to a larger number of younger patients than older patients in their sample. This explanation may be also possible in our study. In addition, from the perspective of the mechanism of renal toxicity caused by acetaminophen, endoplasmic reticulum stress leading to apoptosis may be one of the reasons. In the nervous system, endoplasmic reticulum stress decreases with age (Frakes et al., 2020), meaning that the stress response is stronger in younger people than in older people. Perhaps this trend also exists in the kidneys, where ER stress in young adults is more sensitive or more intense than that in the elderly, and signal transmission is faster and stronger, leading to more severe apoptosis, and ultimately more kidney injury in this age group. Moreover, in the state of chronic overnutrition, the unfolded protein response of the endoplasmic reticulum (UPRER) mechanism of metabolically active cells may be overwhelmed, leading to unresolved ER stress and the deterioration of metabolic tissue (Frakes and Dillin, 2017). Meanwhile, with the development of time, especially in the past decade, the age of obesity gradually decreases (Mohammed et al., 2018). Therefore, the susceptible population of acetaminophen-induced kidney injury is mainly young and middle-aged people, which may be related to uncontrolled ER stress caused by obesity or overnutrition. Besides, young women are indeed more likely to commit suicide using OTC drugs (Hopkins et al., 2020), which may indeed be a contributing factor. However, after we excluded the data of suicide population, we found that the incidence of kidney injury between different genders was still very similar, so in this study, the contribution of suicide might not be very large.

### Time to Onset of Ibuprofen- and APAP-Associated Renal Injury

Based on the FAERS database, the time to onsets of renal events for ibuprofen and APAP mainly occurred within 1 month after administration, but the average onset time of APAP-related kidney injury was significantly shorter than that of ibuprofen-related kidney injury (32.74 vs 115.82 days). Therefore, in a very long period, patients are likely to lead to renal injury for the use of ibuprofen so that the observation of patients who have taken ibuprofen need a longer time to prevent the decline in renal function. On the contrary, the use of acetaminophen is more likely to cause kidney damage in the short term, especially the AKI, which need to pay attention to in the pediatric. Acetaminophen and ibuprofen are the most widely used drugs for the treatment of pain and fever in children, so that as many as 95% of children are exposed to APAP when they are 9 months old (Tan et al., 2020). For example, pain is a common symptom in children with chronic kidney disease and acetaminophen is often used to relieve pain (Reis et al., 2018). In this case, we must be alert to the deterioration of the children’s renal function.

### Fatality due to Ibuprofen- and APAP-Associated Kidney Impairment

Compared with acetaminophen, ibuprofen is more potent, affects the elderly more, and occurs for a longer onset time on average, but the mortality rate is only one-sixth of that of ibuprofen (7.36 vs 44.43%). Maybe this anomaly is also related to their mechanisms. Acetaminophen-associated kidney injury is often accompanied by liver damage (Bessems and Vermeulen, 2001) and AKI correlates with more severe liver injury in patients (Akakpo et al., 2020b). In our research, liver injury as the most reported complication of kidney injury, far exceeds the second-ranked symptom of poisoning. Thus, the dysfunction of two important organs may occur simultaneously in a short period of time, which may overwhelm patients and eventually lead to death. However, ibuprofen-related kidney injury is more likely to occur over a long period of time, giving pharmacists and physicians more time to correct the renal dysfunction. In addition, in our study, the number of suicides using acetaminophen was significantly higher than that of ibuprofen (92 cases vs 48 cases). Based on this, the death rate of APAP-related kidney injury may increase due to the presence of more suicides. However, after excluding the suicide population, the mortality rate of APAP-related kidney injury is still significantly higher than that of ibuprofen, which means that the impact of suicide may not be as large as we thought.

### Limitations

Despite the advantages of real-world research and the data mining techniques in this study, inevitably, there are some limitations to this study. First, in the process of data mining, the imperfection of information, such as incorrect inputs and incomplete reports, may lead to bias in the analysis, which is caused by the FAERS database itself. Second, only a limited number of reports were identified as duplication because they may have different CASEID but overlapping data. When we try to delete some duplicating data based on event_dt, age, sex and reporter_country, a large portion of the report was lost, which may be related to the missing event date, age, and sex. Thus, the method of removing duplicate reports deserves further study. Third, confounding factors are difficult to control. Patients may already have underlying chronic conditions such as cardiovascular disease, or baseline renal insufficiency and renal complications, which can affect renal adverse reactions. Fourth, measures of disproportionality are lacking the incidence denominators, are subject to severe reporting bias, and are not adjusted for confounding (Michel et al., 2017; Raschi et al., 2018), so pharmacovigilance (analysis of spontaneous reporting systems) does not allow for the provision of safety comparisons or evaluation associations among drugs. Therefore, the assumptions generated by disproportionality analyses need to be further validated by more reliable methods. Although these drawbacks above do exist, the FAERS database is able to identify signals of ibuprofen or acetaminophen and kidney injury, and further describe the treatment of ibuprofen and acetaminophen. Our study may provide a new basis for further clinical studies of well-organized ibuprofen- and acetaminophen-associated renal injury.

## Conclusion

In the present study, signals for kidney injury following ibuprofen and acetaminophen in real-world practice were identified based on the FAERS database, and APAP indicated a potential stronger association. There is a significant difference in the time of kidney injury onset after administration, and awareness should be raised for some immediate occurrence following the initial administration. Moreover, APAP-related kidney injury is associated with a higher mortality rate, which may be due to a combination of liver failure and kidney failure. Our study sets the stage for further pharmacovigilance investigation of this matter, and further pharmacoepidemiological studies are needed to test the hypotheses generated by this study.

## Data Availability

The datasets presented in this study can be found in online repositories. The names of the repository/repositories and accession number(s) can be found in the article/Supplementary Material.

## References

[B1] Akakpo J. Y. Ramachandran A. Orhan H. Curry S. C. Rumack B. H. Jaeschke H. (2020a). 4-methylpyrazole Protects against Acetaminophen-Induced Acute Kidney Injury. Toxicol. Appl. Pharmacol. 409, 115317. 10.1016/j.taap.2020.115317 33157119 PMC7888547

[B2] Akakpo J. Y. Ramachandran A. Orhan H. Curry S. C. Rumack B. H. Jaeschke H. (2020b). 4-methylpyrazole Protects against Acetaminophen-Induced Acute Kidney Injury. Toxicol. Appl. Pharmacol. 409, 115317. 10.1016/j.taap.2020.115317 33157119 PMC7888547

[B5] Balachander B. Mondal N. Bhat V. Adhisivam B. Kumar M. Satheesh S. (2020). Comparison of Efficacy of Oral Paracetamol versus Ibuprofen for PDA Closure in Preterms - a Prospective Randomized Clinical Trial. J. Matern. Fetal Neonatal. Med. 33 (9), 1587–1592. 10.1080/14767058.2018.1525354 30227731

[B6] Balestracci A. Ezquer M. Elmo M. E. Molini A. Thorel C. Torrents M. (2015). Ibuprofen-associated Acute Kidney Injury in Dehydrated Children with Acute Gastroenteritis. Pediatr. Nephrol. 30 (10), 1873–1878. 10.1007/s00467-015-3105-7 25895445

[B7] Bessems J. G. Vermeulen N. P. (2001). Paracetamol (Acetaminophen)-induced Toxicity: Molecular and Biochemical Mechanisms, Analogues and Protective Approaches. Crit. Rev. Toxicol. 31 (1), 55–138. 10.1080/20014091111677 11215692

[B8] Carpenter H. M. Mudge G. H. (1981). Acetaminophen Nephrotoxicity: Studies on Renal Acetylation and Deacetylation. J. Pharmacol. Exp. Ther. 218 (1), 161–167. 7241376

[B9] Chen Y. G. Lin C. L. Dai M. S. Chang P. Y. Chen J. H. Huang T. C. (2015). Risk of Acute Kidney Injury and Long-Term Outcome in Patients with Acetaminophen Intoxication: A Nationwide Population-Based Retrospective Cohort Study. Medicine (Baltimore) 94 (46), e2040. 10.1097/MD.0000000000002040 26579812 PMC4652821

[B10] Dixit M. Doan T. Kirschner R. Dixit N. (2010). Significant Acute Kidney Injury Due to Non-steroidal Anti-inflammatory Drugs: Inpatient Setting. Pharmaceuticals (Basel) 3 (4), 1279–1285. 10.3390/ph3041279 27713300 PMC4034033

[B11] Douros A. Bronder E. Klimpel A. Erley C. Garbe E. Kreutz R. (2018). Drug-induced kidney injury: A large case series from the Berlin Case-Control Surveillance Study. Clin. Nephrol. 89 (1), 18–26. 10.5414/CN109212 29035197

[B12] DuMouchel W. (1999). Bayesian Data Mining in Large Frequency Tables, with an Application to the FDA Spontaneous Reporting System. The Am. Statistician 53 (3), 177–190. 10.1080/00031305.1999.10474456

[B13] Eguia L. Materson B. J. (1997). Acetaminophen-related Acute Renal Failure without Fulminant Liver Failure. Pharmacotherapy 17 (2), 363–370. 9085330

[B14] Er R. E. Ulusal Okyay G. Aygencel B Kmaz G. Türko Lu M. Erten Y. (2020). Comparison between RIFLE, AKIN, and KDIGO: Acute Kidney Injury Definition Criteria for Prediction of In-Hospital Mortality in Critically Ill Patients. Iran J. Kidney Dis. 14 (5), 365–372. 32943591

[B15] Evans S. J. Waller P. C. Davis S. (2001). Use of Proportional Reporting Ratios (PRRs) for Signal Generation from Spontaneous Adverse Drug Reaction Reports. Pharmacoepidemiol. Drug Saf. 10 (6), 483–486. 10.1002/pds.677 11828828

[B16] Frakes A. E. Dillin A. (2017). The UPRER: Sensor and Coordinator of Organismal Homeostasis. Mol. Cel 66 (6), 761–771. 10.1016/j.molcel.2017.05.031 28622521

[B3] Fox E. R. Jones V. M. Beckwith M. C. (2012). Acetaminophen Injection: A Review of Clinical Information Including Forms not Available in the United States. J. Pain Palliat. Care Pharmacother. 26 (2), 115–117. 10.3109/15360288.2012.671242 22506845

[B17] Frakes A. E. Metcalf M. G. Tronnes S. U. Bar-Ziv R. Durieux J. Gildea H. K. (2020). Four Glial Cells Regulate ER Stress Resistance and Longevity via Neuropeptide Signaling in C. elegans. Science 367 (6476), 436–440. 10.1126/science.aaz6896 31974253 PMC7357615

[B18] Giménez-Bastida J. A. Boeglin W. E. Boutaud O. Malkowski M. G. Schneider C. (2019). Residual Cyclooxygenase Activity of Aspirin-Acetylated COX-2 Forms 15 R-Prostaglandins that Inhibit Platelet Aggregation. FASEB J. 33 (1), 1033–1041. 10.1096/fj.201801018R 30096040 PMC6355089

[B19] Hauben M. (2003). A Brief Primer on Automated Signal Detection. Ann. Pharmacother. 37 (7-8), 1117–1123. 10.1345/aph.1C515 12841826

[B20] Hauben M. Madigan D. Gerrits C. M. Walsh L. Van Puijenbroek E. P. (2005). The Role of Data Mining in Pharmacovigilance. Expert Opin. Drug Saf. 4 (5), 929–948. 10.1517/14740338.4.5.929 16111454

[B21] Hopkins A. G. Spiller H. A. Kistamgari S. Zhu M. Michaels N. L. Funk A. R. (2020). Suicide-related Over-the-counter Analgesic Exposures Reported to United States Poison Control Centers, 2000-2018. Pharmacoepidemiol. Drug Saf. 29 (9), 1011–1021. 10.1002/pds.4997 32715560

[B22] Kandler K. Jensen M. E. Nilsson J. C. Møller C. H. Steinbrüchel D. A. (2014). Acute Kidney Injury Is Independently Associated with Higher Mortality after Cardiac Surgery. J. Cardiothorac. Vasc. Anesth. 28 (6), 1448–1452. 10.1053/j.jvca.2014.04.019 25440657

[B23] Kolff W. J. (1946). About a Case of Deadly Salicyl Poisoning in a Patient with Nephritis. Geneeskd Gids 24 (12), 139–142. Dutch. 20276436

[B24] Koza Y. (2016). Acute Kidney Injury: Current Concepts and New Insights. J. Inj. Violence Res. 8 (1), 58–62. 10.5249/jivr.v8i1.610 26804946 PMC4729334

[B25] Li Z. (2001). Analysis of 14 Cases of Renal Failure Caused by Long-Term Use of Ibuprofen. Guangxi Med. (06), 1514–1515. (in Chinese).

[B26] Liu Z. Shen Y. (2009). Acute Tonsillitis. Chin. J. Appl. Intern. Med. 29 (05), 469–475. (in Chinese). 10.1007/978-1-84882-596-3_65

[B27] Lorz C. Justo P. Sanz A. Subirá D. Egido J. Ortiz A. (2004). Paracetamol-induced Renal Tubular Injury: a Role for ER Stress. J. Am. Soc. Nephrol. 15 (2), 380–389. 10.1097/01.asn.0000111289.91206.b0 14747384

[B28] Lucas G. N. C. Leitão A. C. C. Alencar R. L. Xavier R. M. F. Daher E. F. Silva Junior G. B. D. (2019). Pathophysiological Aspects of Nephropathy Caused by Non-steroidal Anti-inflammatory Drugs. J. Bras Nefrol 41 (1), 124–130. 10.1590/2175-8239-JBN-2018-0107 30281062 PMC6534025

[B29] McCrae J. C. Morrison E. E. MacIntyre I. M. Dear J. W. Webb D. J. (2018). Long-term Adverse Effects of Paracetamol - a Review. Br. J. Clin. Pharmacol. 84 (10), 2218–2230. 10.1111/bcp.13656 29863746 PMC6138494

[B30] Michel C. Scosyrev E. Petrin M. Schmouder R. (2017). Can Disproportionality Analysis of Post-marketing Case Reports Be Used for Comparison of Drug Safety Profiles? Clin. Drug Investig. 37 (5), 415–422. 10.1007/s40261-017-0503-6 28224371

[B31] Mohammed M. S. Sendra S. Lloret J. Bosch I. (2018). Systems and WBANs for Controlling Obesity. J. Healthc. Eng., 1–21. 10.1155/2018/1564748 PMC5823412 29599941

[B32] Norén G. N. Bate A. Orre R. Edwards I. R. (2006). Extending the Methods Used to Screen the WHO Drug Safety Database towards Analysis of Complex Associations and Improved Accuracy for Rare Events. Stat. Mednov 15 25 (21), 3740–3757. 10.1002/sim.2473 16381072

[B33] Nourjah P. Ahmad S. R. Karwoski C. Willy M. (2006). Estimates of Acetaminophen (Paracetomal)-Associated Overdoses in the United States. Pharmacoepidemiol. Drug Saf. 15 (6), 398–405. 10.1002/pds.1191 16294364

[B34] Ooba N. Kubota K. (2010). Selected Control Events and Reporting Odds Ratio in Signal Detection Methodology. Pharmacoepidemiol. Drug Saf. 19 (11), 1159–1165. 10.1002/pds.2014 20669233

[B35] Pourarian S. Takmil F. Cheriki S. Amoozgar H. (2015). The Effect of Oral High-Dose Ibuprofen on Patent Ductus Arteriosus Closure in Preterm Infants. Am. J. Perinatol 32 (12), 1158–1163. 10.1055/s-0035-1551671 26007314

[B36] Raschi E. Poluzzi E. Salvo F. Pariente A. De Ponti F. Marchesini G. (2018). Pharmacovigilance of Sodium-Glucose Co-transporter-2 Inhibitors: What a Clinician Should Know on Disproportionality Analysis of Spontaneous Reporting Systems. Nutr. Metab. Cardiovasc. Dis. 28 (6), 533–542. 10.1016/j.numecd.2018.02.014 29625780

[B37] Reis A. Luecke C. Davis T. K. Kakajiwala A. (2018). Pain Management in Pediatric Chronic Kidney Disease. J. Pediatr. Pharmacol. Ther. 23 (3), 192–202. 10.5863/1551-6776-23.3.192 29970975 PMC6027978

[B38] Salerno S. N. Liao Y. Jackson W. Greenberg R. G. McKinzie C. J. McCallister A. (2021). Association between Nephrotoxic Drug Combinations and Acute Kidney Injury in the Neonatal Intensive Care Unit. J. Pediatr. 228, 213–219. 10.1016/j.jpeds.2020.08.035 32818481 PMC7752849

[B39] Stern S. T. Bruno M. K. Hennig G. E. Horton R. A. Roberts J. C. Cohen S. D. (2005a). Contribution of Acetaminophen-Cysteine to Acetaminophen Nephrotoxicity in CD-1 Mice: I. Enhancement of Acetaminophen Nephrotoxicity by Acetaminophen-Cysteine. Toxicol. Appl. Pharmacol. 202 (2), 151–159. 10.1016/j.taap.2004.06.030 15629190

[B40] Stern S. T. Bruno M. K. Horton R. A. Hill D. W. Roberts J. C. Cohen S. D. (2005b). Contribution of Acetaminophen-Cysteine to Acetaminophen Nephrotoxicity II. Possible Involvement of the Gamma-Glutamyl Cycle. Toxicol. Appl. Pharmacol. 202 (2), 160–171. 10.1016/j.taap.2004.06.029 15629191

[B41] Su L. Li Y. Xu R. Luo F. Gao Q. Chen R. (2021). Association of Ibuprofen Prescription with Acute Kidney Injury Among Hospitalized Children in China. JAMA Netw. Open 4 (3), e210775. 10.1001/jamanetworkopen.2021.0775 33662136 PMC7933997

[B42] Szarfman A. Machado S. G. O'Neill R. T. (2002). Use of Screening Algorithms and Computer Systems to Efficiently Signal higher-Than-expected Combinations of Drugs and Events in the US FDA's Spontaneous Reports Database. Drug Saf. 25 (6), 381–392. 10.2165/00002018-200225060-00001 12071774

[B43] Szumilas M. (2010). Explaining Odds Ratios. J. Can. Acad. Child. Adolesc. Psychiatry 19 (3), 227–229. 20842279 PMC2938757

[B4] Tallarida R. J. (1982). Tylenol®/Codeine (McNeil). In: TOP 200. New York, NY: Springer.

[B44] Tan E. Braithwaite I. McKinlay C. J. D. Dalziel S. R. (2020). Comparison of Acetaminophen (Paracetamol) with Ibuprofen for Treatment of Fever or Pain in Children Younger Than 2 years: A Systematic Review and Meta-Analysis. JAMA Netw. Open 3 (10), e2022398. 10.1001/jamanetworkopen.2020.22398 33125495 PMC7599455

[B45] Van Driest S. L. Jooste E. H. Shi Y. Choi L. Darghosian L. Hill K. D. (2018). Association between Early Postoperative Acetaminophen Exposure and Acute Kidney Injury in Pediatric Patients Undergoing Cardiac Surgery. JAMA Pediatr. 172 (7), 655–663. 10.1001/jamapediatrics.2018.0614 29799947 PMC6110290

[B46] van Puijenbroek E. P. Bate A. Leufkens H. G. Lindquist M. Orre R. Egberts A. C. (2002). A Comparison of Measures of Disproportionality for Signal Detection in Spontaneous Reporting Systems for Adverse Drug Reactions. Pharmacoepidemiol. Drug Saf. 11 (1), 3–10. 10.1002/pds.668 11998548

[B47] Waring W. S. Jamie H. Leggett G. E. (2010). Delayed Onset of Acute Renal Failure after Significant Paracetamol Overdose: A Case Series. Hum. Exp. Toxicol. 29 (1), 63–68. 10.1177/0960327109350799 19815612

